# The Link between Inflammation, Lipid Derivatives, and Microbiota Metabolites in COVID-19 Patients: Implications on Eating Behaviors and Nutritional Status

**DOI:** 10.3390/ijms25147899

**Published:** 2024-07-19

**Authors:** Viktoria Hawryłkowicz, Beata Stasiewicz, Dominika Maciejewska, Joanna Sołek-Pastuszka, Natalia Komorniak, Karolina Skonieczna-Żydecka, Alexandra Martynova-Van Kley, Ewa Stachowska

**Affiliations:** 1 Department of Human Nutrition and Metabolomics, Pomeranian Medical University, 71-460 Szczecin, Poland; viktoria.hawrylkowicz@pum.edu.pl (V.H.); dominika.maciejewska@pum.edu.pl (D.M.); natalia.komorniak@pum.edu.pl (N.K.); 2 Department of Human Nutrition, The Faculty of Food Science, University of Warmia and Mazury in Olsztyn, Sloneczna 45f, 10-718 Olsztyn, Poland; 3 Department of Anesthesiology and Intensive Care, Pomeranian Medical University, 71-242 Szczecin, Poland; joanna.pastuszka@pum.edu.pl; 4 Department of Biochemical Sciences, Pomeranian Medical University, 71-460 Szczecin, Poland; skonieczna.zydecka@pum.edu.pl; 5 Department of Biology, Stephen F. Austin State University at UT, Nacogdoches, TX 75962, USA; avankley@sfasu.edu

**Keywords:** cytokine, microbiota, lipids, metabolites, nutrition, SARS-CoV-2, COVID-19

## Abstract

Extreme inflammation that continues even after infections can lead to a cytokine storm. In recent times, one of the most common causes of cytokine storm activation has been SARS-CoV-2 infection. A cytokine storm leads to dysregulation and excessive stimulation of the immune system, producing symptoms typical of post-COVID syndrome, including chronic fatigue, shortness of breath, joint pain, trouble concentrating (known as “brain fog”), and even direct organ damage in the heart, lungs, kidneys, and brain. This work summarizes the current knowledge regarding inflammation and the cytokine storm related to SARS-CoV-2 infection. Additionally, changes in lipid metabolism and microbiota composition under the influence of inflammation in COVID-19, along with the possible underlying mechanisms, are described. Finally, this text explores potential health implications related to changes in eating behaviors and nutritional status in COVID-19 patients. Although research on the cytokine storm is still ongoing, there is convincing evidence suggesting that severe immune and inflammatory responses during the acute phase of COVID-19 may lead to long-term health consequences. Understanding these links is key to developing treatment strategies and supporting patients after infection.

## 1. Introduction

The human immune system consists of effector cells that produce cytokines (interleukins, chemokines, interferons, and others). These cytokines play an important role in the immune response. Their anti-inflammatory and pro-inflammatory effects depend on many complex regulatory mechanisms. When this balance is broken, or homeostasis is disturbed, a cascade of complex chemical reactions may be triggered, resulting in the massive release of pro-inflammatory cytokines [1]. This so-called cytokine storm, further intensifying feedback mechanisms, can cause systemic damage and, in extreme cases, multi-organ failure, leading to patient death [1]. Although factors initiating these responses include autoimmune diseases, it has been noted that pathogens such as SARS-CoV-2 (Severe Acute Respiratory Syndrome Coronavirus-2) have played a primary role in recent years [1,2].

In SARS-CoV-2 infection, the virus triggers an excessive immune system reaction, responsible for the severe course of the disease and organ damage, often leading to death. Numerous studies have explored the mechanism of inflammation activation and the associated cytokine storm using the example of Coronavirus disease 2019 (COVID-19), the set of symptoms caused by SARS-CoV-2 infection. The rapid spread of the virus and the frequency of severe cases among infected people prompted the WHO to declare the SARS-CoV-2 coronavirus a public health emergency of international concern (PHEIC) on 30 January 2020, less than two months after the first case detection in Wuhan, Hubei province, China [3,4]. Shortly thereafter, in March 2020, the WHO declared a COVID-19 pandemic [5]. As of today, over 775.7 million confirmed cases of COVID-19 have been reported to the WHO worldwide, including over 7 million deaths. According to the latest data, over 5.47 billion vaccine doses have been administered. About 28% of the world population have received at least one dose of a COVID-19 vaccine [6].

The most common symptoms of COVID-19 include weakness, fever, muscle pain, fatigue, cough, and shortness of breath. In some cases, worsening shortness of breath lead to acute respiratory failure (Acute Respiratory Distress Syndrome, ARDS), requiring intubation and mechanical ventilation. For extreme cases of severe ARDS caused by a massive inflammatory reaction, where conventional mechanical ventilation is insufficient, patients may be qualified for extracorporeal oxygenation therapy (Extracorporeal Membrane Oxygenation, ECMO) [7]. Shortly after the first publications on SARS-CoV-2 appeared, a cytokine storm caused by COVID-19 and the severity of the disease was suggested [7]. The excessive local release of cytokines is believed to be a factor influencing the pathological changes and clinical manifestation of ARDS, specifically the occurrence of viral cytopathic changes in the lung tissue, infiltration of inflammatory cells, and the presence of viral particles [2]. Therefore, severe lung damage in COVID-19 patients is thought to be the result of both direct viral infection and excessive immune system activation.

The pathogenesis of COVID-19 is complex. It can be described by three main pathological processes related to inflammation—local manifestations of inflammation, acute systemic inflammation, and chronic systemic inflammation with low intensity [8,9]. The last one increases with aging and is associated with metabolic syndrome, type 2 diabetes mellitus, and some other chronic diseases [8,9].

## 2. Pathogenesis of the Cytokine Storm in COVID-19

The term “cytokine storm” describes an excessive and uncontrolled immune response. During this event, immune system cells release massive amounts of cytokines, signaling proteins that regulate inflammatory and cellular responses to infection. While crucial for fighting pathogens, excess cytokines can lead to serious tissue damage, multi-organ failure, and even death.

The pathogenesis of the cytokine storm is complex. It involves the loss of regulatory control over the pro-inflammatory cytokines, both at local and systemic levels [10]. This uncontrolled pro-inflammatory response develops rapidly, contributing to high mortality rates. Pro-inflammatory biomarkers of the cytokine storm include interleukins (ILs), IL-1, IL-6, IL-18, IL-31, and IL-33, along with interferon-γ. Additionally, C-reactive protein (CRP) expression has been observed due to IL-6 secretion, with complement activation contributing to tissue damage [1]. Some cytokines, such as IL-4, IL-10, and IL-13, have anti-inflammatory effects [1]. This set of markers during an activated cytokine storm is highly complex, interdependent, and variable. As Fajgenbaum and June [11] described, precisely distinguishing between a pathologically dysregulated inflammatory response and a physiological cytokine response in critical illness can be difficult or even impossible. The SARS-CoV-2 virus is highly transmissible mainly through respiratory droplets larger than 5 μm, and can lead to Severe Acute Respiratory Distress Syndrome, a life-threatening condition, in some infected individuals [5,6,10]. The virus enters cells by binding its Spike protein to the human angiotensin-converting enzyme 2 (ACE2) receptor, found throughout the body, particularly in the respiratory tract [12,13,14]. Recognition of the ACE2 receptor by the Spike protein is a key determinant of tissue tropism and the pathogenesis of this coronavirus [15].

**Figure 1:**
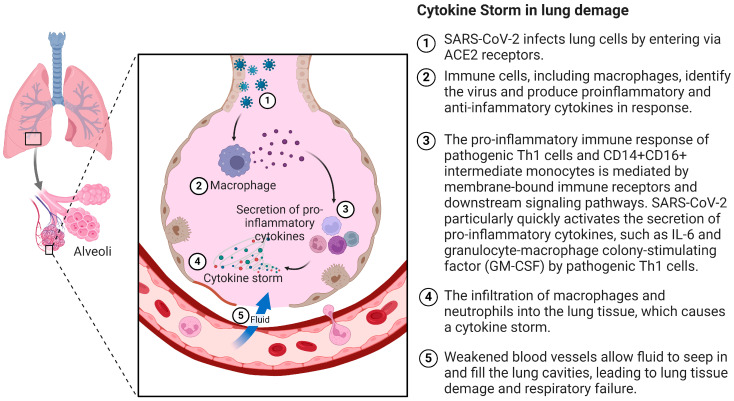
Cytokine storm after entering cells via angiotensin-converting enzyme 2 receptors by SARS-CoV-2. ACE2: angiotensin-converting enzyme 2; Th1: type 1 T helper; GMCSF: granulocyte-macrophage colony stimulating factor. Created with BioRender.com.

Upon entering respiratory epithelial cells, SARS-CoV-2 triggers an immune response characterized by the production of inflammatory cytokines, accompanied by a weak anti-inflammatory interferon (IFN) response [16]. The pro-inflammatory response involves pathogenic Th1 cells and CD14+ CD16+ intermediate monocytes, mediated by membrane-bound immune receptors and downstream signaling pathways. SARS-CoV-2 particularly stimulates the rapid secretion of pro-inflammatory cytokines, such as IL-6 and granulocyte-macrophage colony-stimulating factor (GM-CSF) by pathogenic Th1 cells [2]. Subsequently, macrophages and neutrophils infiltrate the lung tissue, culminating in a cytokine storm [17]. GM-CSF further activates pro-inflammatory CD14+ CD16+, leading to the significant production of IL-6, tumor necrosis factor α (TNF-α), and other cytokines, further amplifying inflammation [18]. A high expression of IL-6 and TNF-α characterizes the cytokine storm in COVID-19. The course of the cytokine storm after entering cells via angiotensin-converting enzyme 2 receptors by SARS-CoV-2 is shown in Figure 1.

The cytokine storm is a hallmark feature of severe COVID-19 and a key contributor to mortality. Evidence suggests a link between dysregulated and excessive cytokine release and rapid deterioration in some patients [10]. While initial symptoms in critically ill and deceased patients may be mild (fever, cough, muscle soreness), their condition worsens quickly. This rapid decline often leads to ARDS, multi-organ failure, and death within a short timeframe. The cytokine storm is considered a major driver of these complications [19]. However, some studies present conflicting data. Research by Ong et al. [20] observed elevated levels of most cytokines (except IL-1) only after a significant decline in respiratory function, challenging the notion of cytokines directly causing respiratory impairment in COVID-19 patients.

In many severe cases of COVID-19, a clear dynamic between cytokine storms and T-cell lymphopenias was observed, which was related to the severity of the coronavirus disease [20]. It was observed that patients with severe COVID-19 had higher plasma levels of C-reactive protein (CRP), IL-6, and IL-8 compared to the patients with a mild–moderate course of the disease [21]. In the study conducted by Huang et al. [7], high expression levels of IL-1B, IL-2, IL-4, IL-6, IL-7, IL-10, IFN-γ, IP-10, TNF-α, and monocyte chemoattractant protein 1 (MCP-1) were observed in COVID-19 patients, but also anti-inflammatory IL-4 and IL-10 secreted by Th2 cells, which is an example of the complexity of the cytokine storm mechanism [7,8,22]. These inflammatory cytokines can trigger activation of the T helper type 1 (Th1) cell response, which precedes activation of the specific response [23]. The available literature describes the difference between the observed concentrations of individual cytokines depending on the severity of the disease. Higher levels of cytokines such as IL-6, IL-10, and TNF-α were observed in patients with severe COVID-19 compared to those with a mild and moderate course of COVID-19 [2]. However, these observations have not been confirmed in other studies [24], as the levels of IL-6, IL-10, and TNF-α were at slightly elevated or within normal ranges. What is more, IL-2 and MCP-1 were elevated in both groups [2]. The number of T lymphocytes (CD4+ T and CD8+ T) was significantly reduced in severely ill COVID-19 patients.

COVID-19 viral infection compared to infection caused by other viruses is characterized by increased pro-inflammatory cytokine levels such as IL-6, IL-1β, and TNF-α. The cytokine storm in patients with COVID19 led to severe lung damage. Another difference may relate to markers of inflammation—patients with COVID-19 often have very high levels of C-reactive protein (CRP), ferritin, and D-dimer, as well as increased neutrophils and decreased lymphocytes [25]. The most differentiating feature is COVID-19’s characteristic pattern of inflammation, which manifests as bilateral frosted-glass lesions on CT scans. In addition, COVID-19 can cause inflammation of the endothelium of blood vessels, leading to microthrombosis and an increased risk of thrombosis [25,26,27].

## 3. Cytokine Storm and Comorbidities

The most frequently reported diseases that significantly increased the prevalence of a severe course of COVID-19 and worse patients’ prognosis have included hypertension (56.6%), obesity (41.7%), diabetes (33.8%), and coronary artery disease (11.1%), which could be related to the development of inflammation [16,25].

A particularly important aspect seems to be the problem of obesity, including sarcopenic obesity, during COVID-19 infection. As known, the condition for maintaining an efficient immune system is to maintain a balance of mechanisms regulating the secretion of cytokines; however, in obesity, these processes are disturbed. This is due to a larger amount of adipose tissue, which is an active endocrine organ. Adipocytes in a state of hypoxia produce pathologically different amounts of adipokines and cytokines, e.g., interleukin-6, tumor necrosis factor (TNF)-α, and an increase in the concentration of C-reactive protein. The study conducted by Ruan et al. showed that IL-6 is an independent risk factor for the development of severe COVID-19, and adipose tissue is one of the main sources of IL-6 secretion [28,29]. Adipose tissue is the site of reactivation of specific cytokines, i.e., TNF-α, IL-1, and IL-6 [30]. Studies conducted among humans have shown that IL-6 production was associated with hyperinflammation [31]. Obese people may experience disorders of the innate and adaptive immune response, disruption of the integrity of lymphoid tissue, and the development and activity of leukocytes [32]. Adipose tissue contains a lot of lymphoid cells, and for this reason, with an excessive mass of this tissue, a disruption of the number of T cells in it is associated with lymphocyte dysfunction and reduces the efficiency of the immune response in defense during infection [33]. Aghili et al. [34] listed several factors regarding the severity of SARS-CoV-2 infection in obese individuals. He mentioned an abnormal immune response due to the disturbed number of macrophages and lymphocytes, high leptin concentration and leptin resistance in the maturation and function of B lymphocytes, and high level of dipeptidyl peptidase-4 [34]. Dipeptidyl peptidase-4 is a transmembrane enzyme of adipose tissue that can inhibit the secretion of cytokines, e.g., IL-6 and IL-10, when its activity is low [34]. Ryan and Caplice presented adipose tissue as an organ that plays the role of a reservoir for the spread of SARS-CoV-2, activation of the immune system, and secretion of cytokines into the body in COVID-19 [35]. Similarly to obesity, other comorbidities are predictors of a worse course of COVID-19. For hospitalized patients with COVID-19 and type 2 diabetes, four key factors have been described: (1) susceptibility to hyperglycemia due to glucocorticoid treatment; (2) less frequent blood sugar monitoring due to reduced contact with healthcare workers to minimize infection risk; (3) patient isolation, which can hinder proper diabetes management; and (4) inappropriate discontinuation of an angiotensin receptor blocker or angiotensin-converting enzyme inhibitors, medications often used to manage diabetes and blood pressure [36].

Interestingly, Leisman’s study found significantly lower levels of IL-6 in severe COVID-19 patients compared to other inflammatory conditions like ARDS, sepsis, and CRS [37]. This suggests a different cytokine release pattern for COVID-19. Excessive cytokine release is a common feature of many viral infections, including influenza, MERS-CoV, dengue, and Ebola. However, the specific cytokine patterns differ for each virus [1].

## 4. Lipid Markers of Inflammation in COVID-19

Lipids and their derivatives can be used to assess the prognosis of the severity of sepsis in patients infected with the SARS-CoV-2 virus. Although very few studies in this area have been published so far, 2023 turned out to be a breakthrough year, with the publication of the results of three studies on lipid derivatives in the course of COVID-19 [38,39,40]. The first work by Hu Meng et al. [38] indicates that the lipid storm caused by infection may be an indicator of poor prognosis in ARDS respiratory failure. When examining patients in the intensive care unit (ICU), it was noted that phospholipases (sPLA2, cPLA2, PLA2G4A, PLA2G2D) and arachidonic acid metabolism products were increased in all patients in the ICU compared to controls. The enzyme sPLA2 was found to be an indicator of COVID-19 severity, correlating with various pro-inflammatory lipid derivatives such as TxA2 and LTE4. It was observed that elevated PGD2 and 12-HETE levels differed specifically in patients with COVID-19, increased lymphopenia, and the severity of the disease [38]. It was also noted that specific lipids, including linoleic acid, were directly associated with SARS-CoV-2 and reflected the severity of COVID-19. In COVID-19, various eicosanoids and lipids were associated with different aspects of the immune response—lipids such as ChoE 18:3, LPC-O-16:0, and PC-O-30:0 in patients were negatively correlated with the activation of specific immune cells and pathway inflammation [38].

**Figure 2:**
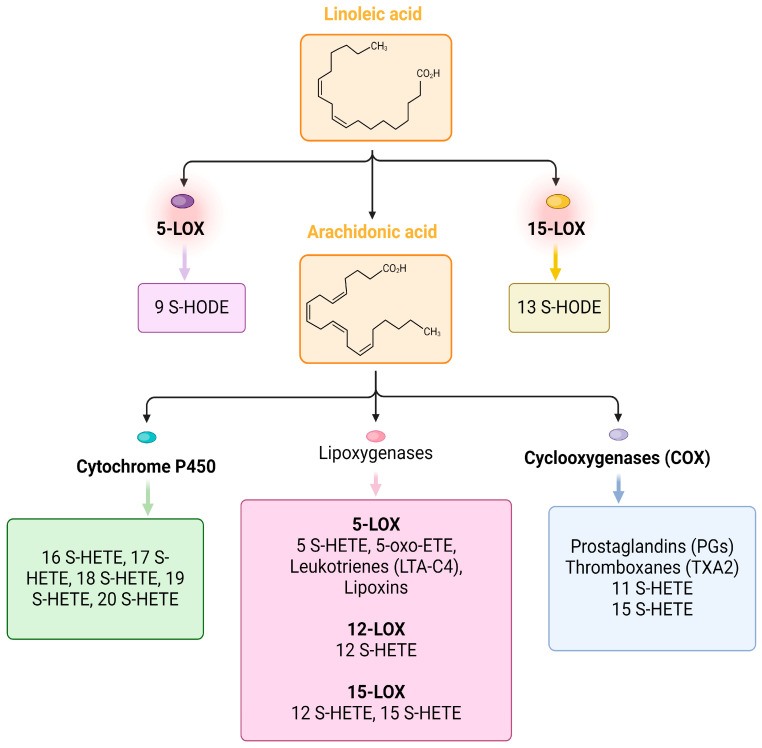
Transformations of selected fatty acid derivatives. Linoleic acid is metabolized by 5-LOX to 9-HODE, and by 15-LOX to 13-HODE. Arachidonic acid can be metabolized by cytochrome P450 enzymes to HETEs including 20-HETE, by COX enzymes to prostaglandins, and by 5-LOX and 12/15-LOX enzymes to lipoxins, leukotrienes, 5-HETE, 12-HETE, 15-HETE, and 5-oxo-ETE. Created with BioRender.com.

The next work published in 2023 [39] indicates a very important role of elevated lipid mediators in the reduction in inflammation. In a study conducted on 56 patients with COVID-19 and 49 control patients with other respiratory diseases, it was found that the levels of cytokines/chemokines (IL-6, CXCL-10, HGF) and some lipid metabolites (TxB2, 11-HETE, 9-HODE, 13-HODE, 5-HETE, 12-HETE, 15-HETE, 14S-HDHA, 17S-HDHA, and 5-oxo ETE) were significantly elevated in patients compared to the controls. In patients with severe COVID-19, anti-SARS-CoV-2 antibodies and pro-inflammatory cytokines and chemokines (IL-6, CXCL-10, and HGF) were significantly elevated. Lipid mediators involved in the reduction/resolution of inflammation (5-HETE, 11-HETE, and 5-oxoETE) were significantly elevated in mild/moderate disease [39]. The transformations of selected fatty acid derivatives are illustrated in Figure 2. Interestingly, patients with severe disease had significantly increased levels of pro-inflammatory cytokines/chemokines and antibodies against SARS-CoV-2. In patients with mild disease, it was noticed that lipid metabolites such as 5-HETE, 11-HETE, and 5-oxoETE, involved in reducing inflammation, were increased in the blood.

In the third study, also published in 2023 [40], liquid chromatography–mass spectrometry (LC-MS) methods were used to determine the difference in the severity of inflammation, and the measurements were performed in liquefied breath samples (EBC) from COVID-19 patients and control groups. Researchers identified significant changes in the levels of inflammatory oxylipins, including prostaglandins A2 and D2, LXA4, and 5-HETE. Differences were detected in the concentration of oxylipins, which are associated with inflammatory processes and the activation of oxidative stress. Higher concentrations of pro-inflammatory lipids in samples have been observed in COVID-19 patients [40].

## 5. Cytokine Storm and Microbiota Metabolites

Severe infections, including COVID-19, are associated with unfavorable changes in the composition and number of microorganisms inhabiting the human digestive tract [41,42]. The human microbiome is important for the homeostasis and proper function of the host’s organs through specific, more or less complex interactions, which have been widely described [43,44]. Particularly important in the context of this work is the impact of the microbiome on immunomodulation; the function of the intestinal barrier, i.e., an element of the immune system at the intestinal level; and the fact that the composition of the microbiota depends on the tissue area and the pH in which it lives [14,43]. Humans harbor a unique gut microbiome shaped by various factors, including age, location, ethnicity, race, gender, and diet [45]. Due to this vast individual variability in the composition and abundance of specific bacterial species, a single “correct” gut microbiome composition cannot be precisely defined. However, significant changes in the microbiome associated with an increase in harmful pathogens are identified as dysbiosis.

SARS-CoV-2 infection was associated with the observed unfavorable changes in the microbiota of COVID-19 patients, consisting of a reduction in the number of commensals in favor of opportunistic pathogens and the resulting deterioration of the intestinal barrier function [45,46,47]. Dysbiosis is observed in obesity, inflammatory bowel disease (IBD), skin psoriasis, and inflammatory joint disease, as well as influenza and viral liver disease caused by HBV infection or HIV infection [48,49,50,51,52]. Also in the case of COVID-19 patients, dysbiosis features of the oral and intestinal microbiome have been demonstrated during the ongoing viral infection as well as during the convalescence period [45,46,47,53,54,55,56]. This is not only due to the fact of infection but also to the location of ACE2 receptors, which are also located in the gastrointestinal tract. The virus using ACE2 receptors might lead to local inflammation, intestinal symptoms, and dysbiosis [57]. Yeoh et al. [41] reported a treatment-independent reduction in the population of immunomodulatory bacteria from *Bifidobacteria*, *Eubacterium rectale*, and *Faecalibacterium prausnitzii* in patients hospitalized with COVID-19, and dysbiosis persisted even after recovery. Yeoh et al. [41] noticed that specific species of intestinal microbiota in high or low abundance in study participants correlated with the concentration of inflammatory markers in plasma, i.e., IL-10, TNF-α, CRP, etc.

The gut microbiome and the immune system are linked by a complex communication pathway known as the gut–lung axis [58]. This link is based on mutual influence. For example, the state during infection influences the composition and diversity of the microbiome, which influences the immune response in the event of infection [58]. A meta-analysis comparing the microbiome of COVID-19 patients and uninfected patients showed the loss of beneficial intestinal bacteria, i.e., *Fusicatenibacter*, *Lachnospiraceae* from the NK4A316 group, *Lachnoclostridium, Blautia*, and *Roseburia*, and an increase in the number of opportunistic bacteria *Streptococcus*, *Rothia*, and *Actinomyces* in patients infected with SARS-CoV-2 [42]. Reductions in *Firmicutes* diversity and abundance have been reported in respiratory infections [58]. This is an interesting observation because these bacteria are responsible for strengthening and maintaining the integrity and thus the function of the mucous membrane. It also shows immunomodulating properties, protecting against pathogen invasion. The mechanisms of immunomodulation are the production of specific bacterial metabolites, e.g., short-chain fatty acids (SCFAs) and antimicrobial peptides (AMPs) [59].

### 5.1. Short-Chain Fatty Acids (SCFAs)

Commensal bacteria ferment plant fiber, producing short-chain fatty acids (SCFAs) like acetate, propionate, and butyrate. These SCFAs exert anti-inflammatory effects by binding to immune cell receptors. They also reduce pro-inflammatory cytokine production while favoring anti-inflammatory ones, and even enhance the activity of CD8+ T lymphocytes [60,61]. Studies suggest a decrease in SCFA-producing bacteria in COVID-19 patients with dysbiosis [62,63]. Given the crucial role of SCFAs in the gut–lung axis, their decline is associated with generalized inflammation. In uninfected individuals, it may even elevate the risk of lung infection [64,65,66]. Notably, acetate can enter the bloodstream and activate a receptor called the G-protein-coupled receptor. This activation improves the response to type I interferon (IFN) and boosts IFN-stimulated gene expression, ultimately bolstering the body’s defenses against infection [51]. Interestingly, research suggests that SCFAs, particularly butyrate, may play a role in preventing the development of cytokine storms and systemic damage in COVID-19 patients [67].

Several factors contribute to changes in the gut microbiome during respiratory infections. These include the release of pro-inflammatory cytokines, reduced energy intake due to appetite loss (or lack of taste and smell in COVID-19), and protein insufficiency. Additionally, weight loss caused by calorie deficits or hypercatabolism during illness can be a contributing factor. While reducing energy intake can impact the gut microbiota, it is important to remember that a decrease in plant fiber consumption will also lead to unfavorable changes. This, in turn, reduces the production of SCFAs and other beneficial bacterial metabolites [39,40].

### 5.2. Deoxycholic Acid (DCA) and Lithocholic Acid (LCA)

The human gut microbiome also plays a role in processing bile acids into secondary forms like deoxycholic acid (DCA) and lithocholic acid (LCA). These transformed bile acids then act as signaling molecules (ligands) for specific receptors in various organs, including the liver (hepatocytes), epithelial and endothelial cells, pancreas, brain, and lymphoid tissues. Through these interactions, they exert immunomodulatory effects [68,69]. However, in COVID-19 patients, intestinal dysbiosis and diarrhea may disrupt the reabsorption of DCA and LCA, potentially posing a problem. Studies have shown the presence of secondary bile acids in the feces of critically ill COVID-19 patients [70]. The influence of high concentrations of secondary bile acids in the intestinal lumen on the deterioration of the intestinal barrier and increased permeability has also been described [71].

### 5.3. Antimicrobial Peptides (AMPs)

Antimicrobial peptides (AMPs) can be divided into groups in terms of action. Among them, we can distinguish proteins with antibacterial, antifungal, and antiviral activities [59]. The action of antiviral proteins is based on (1) inhibiting virus attachment and virus cell membrane fusion, (2) destroying the virus envelope, or (3) inhibiting virus replication. The most well-described AMPs obtained from microorganisms like bacteria and fungi are nisin, gramicidin, and defensin produced by Lactococcus lactis, Bacillus subtilis, and Bacillus brevis [59]. There are not many scientific reports on the use of specific microbiota-derived AMPs as a useful tool in the treatment and prevention of SARS-CoV-2 infection. However, molecular docking analysis indicated that peptides were employed to disrupt the interaction between COVID-19 and ACE2 to inhibit COVID-19 entrance into cells [72]. It was mentioned before that some animal-derived and synthetic AMPs might be useful in terms of SARS-CoV-2 infection. Among these, there are defensins [73]. The lectin-like human α-defensin 5 (HD5) binds to the ligand-binding domain of ACE2 glycoproteins and inhibits entry into the cells by SARS-CoV-2 virus [73]. This interference between viral glycoproteins and receptors of the cell activates the adaptive immune antigen-presenting phagocytes cells and disrupts cellular replication by the virus [73]. The increased expression of hBD2, hBD3, elafin, adrenomedullin, cathelicidin (LL-37), IL-6, CXCL8, CXCL1, and CCL2 enhanced epithelial maturation and thus the epithelial barrier. This mechanism depended on early colonizing reconstituted human gingiva by commensal bacteria such as Granulicatella, Veillonella, and Streptococcus and resulted in a greater organization of the epithelial layer and barrier against infection [59]. The lipopeptide EK1C4, derived from EK1, is the most effective fusion inhibitor against COVID-19 Spike protein-mediated membrane fusion [74]. Two AMPs from the non-structural protein nsp10 of SARS-CoV, K12, and K29 can inhibit SARS-CoV replication.

### 5.4. Tryptophan Derivatives

The cytokine storm in COVID-19 also disrupts tryptophan metabolism. This amino acid can be metabolized through the kynurenine and serotonin pathways, but the gut microbiome offers another route. This microbial pathway produces various metabolites like tryptamine, indole-3-ethanol (IE), and indole-3-propionic acid (IPA) [75]. These metabolites are believed to exert anti-inflammatory effects by promoting the production of antimicrobial peptides (AMPs) via increased IL-22 expression and reducing the secretion of pro-inflammatory cytokines like IL-6 and IL-1 in intestinal epithelial cells [76,77]. However, SARS-CoV-2 infection disrupts this process. The virus utilizes the ACE2 receptor, which is also present on the surface of small intestine cells in COVID-19 patients. This interaction downregulates a specific protein called SLC6A19 (Solute Carrier Family 6 Member 19), hindering tryptophan uptake by these cells [40,41]. Reduced SLC6A19 activity is associated with lower AMP production in the small intestine, potentially increasing susceptibility to infection and further disrupting the gut microbiome [78,79]. Consequently, due to this blocked absorption, tryptophan levels in the serum of COVID-19 patients are higher compared to healthy individuals [39,40].

### 5.5. Desaminotyrosine

Beyond SCFA tryptophan derivatives and other AMPs, desaminotyrosine is also mentioned as an immunomodulatory bacterial metabolite [80,81,82]. This molecule originates from the breakdown of flavonoids and amino acids. Specifically, Clostridium orbiscindens bacteria generate desaminotyrosine during the digestion of plant flavonoids. Notably, desaminotyrosine can enter the bloodstream and enhance type I interferon (IFN) signaling. This, in turn, stimulates the innate immune system in the lungs, bolstering the body’s defense against influenza infection within the framework of the gut–lung axis [82]. The mechanism of protection of the host by this microbial metabolite is due to priming the amplification loop of type I IFN signaling. Desaminotyrosine demonstrated the ability to treat viral infection in mice by increasing the expression of multiple type I IFN-stimulated genes in the lungs by increasing the production of type I IFN in response to viruses [82].

## 6. Eating Behavior in the Pandemic: Implications on the Immune System

The COVID-19 pandemic has changed the lives of populations around the world. Forced stays at home during the COVID-19 lockdown limiting access to healthcare, remote work, monotony, social distancing, stress, and more time for cooking in their homes have directly influenced the eating behaviors of various societies affected by the epidemic [83].

Johnson et al., based on the analysis of 71 studies, concluded that eating behaviors in most cases remained unchanged during the pandemic [84]. In turn, Grant et al. [85], based on the eating behavior of Italians (*n* = 2678 respondents), noted that, during the pandemic, four different consumer attitudes could be distinguished during the lockdown in response to existing conditions: 1. the “healthy eaters”; 2. the “less eaters”—respondents who decreased overall food consumption; 3. the “usual eaters”—those who did not change their food consumption pattern; and 4. the “more eaters”—people who increased food consumption in all food categories. The “healthy eaters” group was characterized by the increased consumption of legumes, whole-grain cereals, non-whole pasta, rice, and nuts, but also sweets and pastries [85]. Unfortunately, all groups, regardless of consumption patterns, often reported low levels of physical activity [85].

In the study by Johnson et al. [84] where food intake changed, an increase in consumption was observed. Of those surveyed, 44.9% reported no changes in the amount of food they consumed during the COVID-19 pandemic, and 31% reported an increase in food consumption. Similarly, the frequency of meals and snacks consumed was essentially unchanged, with the next highest response being an increase in the number of meals and snacks consumed [84]. In terms of changes in the types of foods consumed during the pandemic, consumption in most food groups remained the same, but trends varied by food category and region. For fruit and vegetables, legumes, white bread/pasta, home-baked goods, and snacks (general as well as sweet, savory, and salty), participants reported unchanged or increased intake. Reduced intake has been reported for some vegetables, such as dark green leafy vegetables. For dark bread/cereals, meat (including red meat and processed meats), seafood/fish, frozen foods, and fast food, participants typically reported no change or reduced intake [84].

In the Spanish population, a lower consumption of drinks, slightly higher consumption of eggs and red meat (above the recommended range), and a significant increase in consumption of plant-based foods such as nuts, pasta, rice, or processed vegetables were observed, but still below the recommended range [86]. However, in the Chinese population, the frequency of consumption of fresh vegetables and fruit, rice, poultry, meat, and soy products decreased [87]. In turn, in the population of teenagers from Brazil, Chile, Colombia, Spain, and Italy, the frequency of eating vegetables, legumes, and fruit increased [83].

Many of these increases and decreases can be explained, at least in part, by the limited access to restaurants during COVID-19 lockdowns. In fact, there was an increased consumption of home-cooked meals overall and of specific types of home-cooked foods measured separately, such as pizza and sweets. However, purchasing and consumption behaviors may have changed more for certain food categories due to perceptions of COVID-19 and its transmission. People concerned about COVID-19 infection may be less likely to buy fruits and vegetables with porous and/or edible skin [84]. The results also show that binge eating, uncontrolled eating, and overeating have increased during the COVID-19 pandemic. Various results regarding restrictive eating have been observed depending on the region [84].

In the context of energy value, a higher energy intake and higher consumption of low-nutrient-density foods were recorded compared to the period before the pandemic [88]. Up to 539 kcal more than the daily recommended energy requirement was consumed [88], which, compared to the same period in 2019, resulted in an increase in daily consumption by 6%. This was associated with a greater food intake of main meals and snacks, especially in overweight and obese populations [89]. The frequency and volume of consumption of certain products have changed [86,90].

A change in lifestyle, including inappropriate eating behavior, was observed primarily in overweight and obese people [91]. In addition to increasing the frequency of eating and snacking, these people reported a lower frequency of consumption of fruit, vegetables, and legumes, and a higher consumption of dairy products, meat, and fast food [92]. Additionally, there was an increase in alcohol consumption (by 15%) and an increase in the frequency of tobacco smoking [92].

Eating behaviors are linked to the development of chronic diseases, and as new evidence shows, to susceptibility to COVID-19 [93]. Eating behaviors are an important determinant of the functioning of the immune system [94,95,96]. On the one hand, nutrients and phytochemicals may have immunomodulatory effects; on the other hand, their deficits may increase the host’s susceptibility to viral infection and its more severe course [93,94,96]. Eating behaviors directly influence body weight and composition, which in turn affect physiological processes [97].

## 7. Bioactive Food Compounds, Immune System, and COVID-19

A balanced diet based especially on fresh vegetables, fruits, nuts, fish, and olive oil provides all the necessary nutrients and thus plays an important role in the immune system [93]. These immunomodulatory effects result from the fact that many food compounds have additional, biologically active (bioactive) properties beyond nutritional ones. Many nutrients modulate the immune system response, for example, the minerals Zn, Se, Fe, Mg, and Cu, as well as vitamin D, vitamin A, B vitamins (folic acid, vitamins B6, and B12), vitamin C, and vitamin E [93,96].

During the COVID-19 pandemic, the increased use of vitamins and mineral supplements, particularly vitamin C, vitamin D, zinc, and selenium, was observed [98]. Many studies evaluated the efficiency of intervention of some vitamins and minerals supplementation in COVID-19 infections [99,100,101,102,103,104,105,106,107,108,109]. Since viral infections increase oxidative stress, these studies focused mainly on antioxidant nutrients such as vitamins C and D, zinc and selenium, or multivitamins also containing flavonoids such as quercetin and bromelain. The available studies that evaluated the role of nutrients in COVID-19 management focused on supplementations and did not assess dietary nutrient intake [99,100,101,102,103,104,105,106,107,108,109].

Preliminary studies have shown promising results of some dietary supplement use in patients with COVID-19 [106,107]. However, the latest randomized clinical trials and meta-analyses of 37 RCTs showed no clinically or statistically significant effects of the administration of multivitamin supplements of vitamin C and vitamin D combined with vitamin A, vitamin B, vitamin K2, and zinc on improving overall health and reducing symptom burden in COVID-19 patients [99,100,104]. This heterogeneity of results could have been caused by the high variety in recruitment (sample size, different populations, age, sex, hospitalized or non-hospitalized patients, COVID-19 severity, treatment strategies) and dietary supplement administration (one or combined supplements, dosage, form, route, frequency and duration administration) among COVID-19 patients. Benefits of the regular use of vitamin C and vitamin D were reported only in decreasing all-cause mortality among patients with COVID-19 [103,104]. These findings suggest the presence of a positive effect of vitamin C and D on several inflammatory mechanisms induced by SARS-CoV-2.

Vitamin C and vitamin D play a crucial role in the immune system by regulating the growth and activity of innate and adaptive immune cells, and the production of antibodies [101,102,103,104]. In clinical trials, vitamin D administration in hospitalized COVID-19 patients resulted in a beneficial increase in the level of natural killer (NK) neutrophil cells, and the neutrophil-to-lymphocyte ratio [106,108]. Vitamin D improves the physical barrier against viruses and stimulates the production of antimicrobial peptides. The sufficient status of vitamin D enables the activation of vitamin D through the expression of its receptor and enzyme 1-α-hydroxylase in macrophages during pathogen invasion [105]. Intracellular vitamin C increased chemotaxis, phagocytosis, and microbial killing by neutrophils. Furthermore, vitamin C facilitates the activation of cytotoxic T cells (CTL) by promoting T helper 1 (Th1) skewing. This vitamin is responsible for upregulating the expression of interferon type 1, and defense against infection [98]. Vitamin C and Vitamin D as robust antioxidants can reduce oxidative stress, which is considered a key point in the pathophysiology of COVID-19 [100]. Both vitamins can reduce the risk of a cytokine storm by decreasing reactive oxygen species and pro-inflammatory cytokines including IL-6 [102]. Moreover, vitamin C influences anti-inflammation through the activation of the nuclear transcription factor kappa B (NF-κB). Other vitamins, including Vitamin A, also have anti-inflammatory and immunomodulatory properties by intensifying immune cell activation including B and T cells [100].

Among the minerals, the greatest interest in the context of impact on the immune system is focused on selenium and zinc. Selenium in the form of selenocysteine is part of the catalytic site of peroxidases, which play a role in redox homeostasis and antioxidant defense [101,105]. Deficiencies of selenium and selenoproteins increase oxidative stress, which can lead to viral mutation, and increased pathogenicity. Similarly, the deficiency of zinc and copper may lead to negative changes in the number and function of immune cells, and thus to the dysregulation of immune homeostasis [100].

Macronutrients also play a role in the body’s immune functions. Protein intake plays a key role in cellular immunity. In the case of protein malnutrition, characteristic of populations from low-income countries, impaired immune functions of macrophages, neutrophils, and natural killer (NK) cells are observed [105]. This protein deficiency leads to decreased levels of T-lymphocytes and then to increased susceptibility to viral infections. Protein and amino acids are involved in maintaining homeostasis; hence, adequate protein intake is important in reducing infectious complications in the critically ill, including patients with COVID-19 [105].

Fats have not been often evaluated in research in the context of their effects on the immune system and COVID-19. Mazidimoradi et al. [109] reported decreased levels of polyunsaturated omega-3 fatty acids (PUFAs n-3) among patients with COVID-19. They also found that PUFAs n-3 can reduce the severity of COVID-19 and the chance of hospitalization and mechanical ventilation. These data support the important role of PUFAs n-3 in regulating immune pathways through its anti-inflammatory properties [109]. On the other hand, the elevated levels of PUFAs mainly from supplements should be avoided due to their susceptibility to oxidation. Additionally, Calder et al. [93] suggested that micronutrients combined with n-3 acids (eicosapentaenoic acid and docosahexaenoic acid) can enhance the immune system’s response to viruses.

## 8. Obese Patients with COVID-19: Risk Factor for Worse Prognosis

Obese patients have a more frequent severe clinical course of COVID-19 and a higher mortality rate. Compared to patients with normal body weight, obese individuals have a higher risk of COVID-19 (over 46%), hospitalization (113%), admission to the ICU (74%), and risk of death (>48%). In a study by Cuerda et al. [110], 60.2% of COVID-19 patients admitted to the intensive care unit were obese (BMI ≥ 30 kg/m^2^). A higher BMI value was associated with increased inflammation, muscle damage, and in-hospital mortality within the first 28 days [110]. The fat tissue mass and its body distribution can play a key role in prognosis of the disease. It was suggested that visceral adipose tissue (VAT) may be a better predictor of COVID-19 severity than BMI [111]. Huang et al. in a meta-analysis indicated VAT as a risk factor for hospitalization, admission to the intensive care unit, and invasive mechanical ventilation [112].

Besutti et al. showed that total body fat, VAT, and muscle density are associated with the risk of hospitalization, mechanical ventilation, or death [113]. Patients with sarcopenia are more susceptible to infection, weak immune response, and a greater predisposition to lipotoxicity, dysregulated metabolism, and ongoing inflammation [114]. In the study by Wierdsma et al. [115], excess body weight was diagnosed in 67% of patients with COVID-19 admitted to the hospital (BMI > 25 kg/m^2^), and 73% had a high risk of developing sarcopenia. Most of these patients required treatment in the intensive care unit [115]. Critically ill patients with severe COVID-19 with low-quality muscle and higher intermuscular adipose tissue (IMAT) were diagnosed with higher mortality and greater inflammation. Additionally, they had greater muscle damage during hospitalization compared to people with low muscle fat [116].

## 9. Malnutrition in Patients with COVID-19: A Result of Hospitalization

Malnutrition during hospitalization affects 20–50% of patients [117,118]. In the case of the elderly, this percentage is much higher and may be up to 90%. It is worth noting that 49% of malnourished patients who stayed in the hospital for more than 1 week maintain or worsen their nutritional status [118]. Malnutrition can be defined as a condition resulting from insufficient food intake, leading to a decrease in lean body mass and worsening psychophysical functioning [119]. It may be the result of starvation, old age (>80 years), or disease and pharmacological treatment (e.g., proton pump inhibitors) [120].

One of the diseases that significantly increase the risk of malnutrition is COVID-19. In the study by Cuerda et al. [110], the percentage of obese patients with COVID-19 (BMI ≥ 30 kg/m^2^) decreased from 60.2% to 23.3% during and after intensive-care hospital admission, respectively, indicating an average weight loss of 16.6%. Regarding the MUST (Malnutrition Universal Screening Tool) and SARC-F scores, 83.5% and 86.9% of patients, respectively, were considered at high risk of malnutrition and sarcopenia at the time of hospital discharge [110]. Wierdsma et al. [115] indicated that 21% of COVID-19 patients during hospitalization were diagnosed with malnutrition based on significant weight loss (>5 kg). A meta-analysis by Feng et al. [117] showed that the risk of malnutrition was higher among people hospitalized in the intensive care unit due to COVID-19 compared to patients in the general ward, and amounted to 92.2% and 70.7%, respectively. This may suggest that malnutrition poses a significant threat to patients with severe symptoms in the course of COVID-19 [117]. This is a particularly important observation because the COVID-19 pandemic has caused a sharp increase in hospitalizations, and the development of malnutrition may generate many undesirable reactions, i.e., rapid muscle loss, a weakened immune response, a longer hospital stay, and even higher hospital mortality [121,122,123].

Systemic inflammation, immobilization, hypoxemia, and malnutrition may cause a significant reduction in muscle mass, along with a progressive loss of muscle strength and function, leading to the development of sarcopenia in people who have experienced severe COVID-19 [124]. Additionally, there are many factors reported by patients that may have a significant impact on the nutritional status, i.e., decreased or loss of appetite, a feeling of fullness, changes/loss of taste/smell, fatigue, nausea, vomiting, problems with biting, pain in the mouth, and defecation disorders [125]. Moreover, these symptoms may continue even many months after the patient’s discharge from the hospital. Therefore, it seems crucial to assess the nutritional status of COVID-19 patients, plan and implement appropriate nutritional management, and monitor its effects on an ongoing basis, both during the hospital stay and after discharge to their home, as an important element of recovery [125,126,127]. Moreover, it turns out that the assessment of nutritional status using the PNI (the Prognostic Nutritional Index), CONUT (the Controlling Nutritional Status Score), NUTRIC (the Nutrition Risk in Critically Ill), or mNUTRIC (the modified NUTRIC) scale effectively predicts in-hospital mortality in patients with severe COVID-19 in the intensive care unit. Patients with a higher risk of malnutrition expressed using the above-mentioned scales have significantly worse treatment results. Additionally, these scales turned out to have similar prognostic values to the APACHE II (Acute Physiology and Chronic Health Evaluation II) and SOFA (Sepsis-related Organ Failure Assessment) scales, which are often used in intensive care [128].

In conclusion, COVID-19 and sarcopenia are linked in a dangerous vicious circle, and aging, immobilization, bed rest, and low levels of physical activity further exacerbate this effect [114,115]. Undoubtedly, nutritional status may influence the course of viral infections by modulating the functions of the immune system. Therefore, it influences the duration, severity, and overall outcome of the disease. On the other hand, the infection itself is a risk factor for deterioration of the nutritional status [114,115].

A properly composed diet can have a beneficial effect on the inflammatory process and, at the same time, reduce the risk of severe disease and mortality due to COVID-19 [129,130]. Research conducted so far shows that the patient’s nutritional status does not determine COVID-19 infection, but deficiencies of certain nutrients influence the course of the disease and are a prognostic factor. This rule applies primarily to deficiencies of selenium and vitamins D and C, as well as a suboptimal intake of iron and vitamin B12 [131]. This allows us to conclude that deficits of these important nutrients may potentially promote the spread of COVID-19 by reducing resistance to infection and reinfection [131].

## Data Availability

Data are contained within the article.
